# Desiccation to DNA: Bulk DNA Metabarcoding of Macroinvertebrate Egg Banks via Sugar‐Flotation Enhances Biodiversity Surveys of Dry Salt Lakes

**DOI:** 10.1002/ece3.74229

**Published:** 2026-08-21

**Authors:** Angus D'Arcy Lawrie, Pia Dethlefsen, Mahabubur Rahman, Christopher Hofmeester, Jessica Delaney, Matthew A. Campbell, Joshua P. Newton, Marina Elisa de Oliveira, Joel Huey, Morten E. Allentoft, Mattia Saccò

**Affiliations:** ^1^ Trace and Environmental DNA (TrEnD) Lab, School of Molecular and Life Sciences Curtin University Perth Western Australia Australia; ^2^ Biologic Environmental Osborne Park Western Australia Australia; ^3^ Section for GeoGenetics Globe Institute University of Copenhagen Copenhagen Denmark; ^4^ Subterranean Research and Groundwater Ecology (SuRGE) Group, Trace and Environmental DNA (TrEnD) Lab, School of Molecular and Life Sciences Curtin University Perth Western Australia Australia; ^5^ Laboratoire de Biologie des Organismes et des Écosystèmes Aquatiques‐BOREA Muséum National d'Histoire naturelle, SU, CNRS, IRD, UA Paris France; ^6^ Department of Chemistry, Life Sciences and Environmental Sustainability University of Parma Parma Italy

**Keywords:** aquatic macroinvertebrates, crustaceans, environmental DNA, extremophiles, salt lakes

## Abstract

Ephemerally inundated salt lakes in arid regions support unique aquatic macroinvertebrate communities that typically persist through dry periods as resistant resting‐stages (e.g., spores, eggs). Salt lakes represent a large proportion of inland lentic habitats on the Australian continent, many occurring in remote arid areas where filling events are infrequent and unpredictable. Currently, biodiversity surveys of dry salt lakes rely on artificial rehydration of sediments to stimulate emergence of resident macroinvertebrate taxa. However, egg banks contain cryptic assemblages with poorly understood hatching requirements that may fail to emerge under artificial rehydration conditions and underestimate biodiversity. DNA metabarcoding of resting eggs may offer a complementary approach by enabling the detection of taxa independent of emergence. We conducted two experiments using sediments from an ephemeral salt lake in central Western Australia, applying two metabarcoding assays (fwh and Crust16S) targeting aquatic invertebrates. Experiment 1 compared a sugar‐flotation pre‐extraction method versus a standard sediment DNA extraction protocol. Experiment 2 investigated how sediment weight (50, 250, 500 and 1000 g) subjected to sugar‐flotation influenced the biodiversity detected. Sugar‐flotation vastly outperformed standard DNA extraction, with DNA sequences from target taxa almost exclusively recovered when the sugar‐flotation step was applied. Species richness increased significantly from 50 to 250 g and continued increasing until 1000 g although pairwise comparisons among the 250, 500 and 1000 g samples were not significant. DNA metabarcoding results from the 1000 g treatment were compared with collections of taxa recovered from a contemporaneous rehydration trial. DNA metabarcoding detected more taxa, including seven unique taxa, whereas only one taxon was exclusive to rehydration trials. Our findings indicate that bulk metabarcoding of egg banks isolated by sugar‐flotation is an effective method for documenting aquatic macroinvertebrate communities in dry salt lakes. This represents a valuable addition to monitoring programmes, enhancing effective management and conservation of these important ecosystems.

## Graphical abstract

Ephemeral salt lakes in arid Australia contain hidden macroinvertebrate biodiversity in sediment egg banks, but traditional artificial rehydration surveys may miss taxa with specific or poorly understood hatching requirements. This study found that sugar‐flotation followed by DNA metabarcoding greatly improved detection of resting‐stage taxa compared with standard sediment DNA extraction, with larger sediment samples generally recovering more diversity. Overall, metabarcoding of egg banks detected more taxa than rehydration trials, showing that this approach is a valuable complementary tool for biodiversity monitoring and conservation of dry salt lake ecosystems.

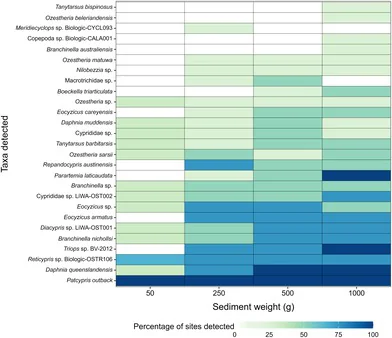

## 1 Introduction

Most waterbodies in arid regions hold water on a seasonal or episodic basis, yet they often support a diverse and unique aquatic fauna (Timms 2008). These environments are dominated by aquatic macroinvertebrates with desiccation‐resistant life stages that can persist through dry periods and may remain viable for decades or longer (Radzikowski 2013). Such diapausic and quiescent strategies typically consist of eggs or larval/nauplii stages, which form ‘egg banks’ that allow populations to re‐establish after re‐inundation (Pinceel et al. 2021). Egg banks in temporary waterbodies are dominated by crustaceans, including anostracans, cladocerans, ostracods, diplostracans and copepods (Lawrie et al. 2021; Rahman et al. 2022; Schwentner et al. 2015; Timms 2014).

Salt lakes (non‐marine enclosed bodies of water with salinities greater than 3 g/L; Bayly and Williams 1966) account for almost half (~44%) of the volume of inland waters on the planet (Messager et al. 2016) and occur on every continent (Hammer 1986). Indeed, salt lakes constitute the majority of the inland lentic waters of Australia and are particularly conspicuous in the arid zone, where vast salt lake playas have formed in the residual depressions of paleo‐drainage channels (Timms 2008; Timms et al. 2006; van de Graaff 1977). A community of specialised terrestrial macroinvertebrates has evolved to occupy these dry lake beds (Framenau and Hudson 2017; López‐López et al. 2016; Schultheiss et al. 2013) as filling events are rare and unpredictable, typically limited to rainfall from ex‐tropical cyclones (Gregory et al. 2009). After inundation, the emergence of the dormant aquatic macroinvertebrate taxa transforms these salt lakes into enormously productive ecosystems that can attract thousands of waterfowl, some of which are dependent on these remote salt lakes for breeding (Pedler et al. 2014; Pedler et al. 2018).

Relative to salt‐lake environments globally (Saccò et al. 2021), a diverse and overwhelmingly endemic suite of aquatic macroinvertebrate fauna occurs in Australian salt lakes (Lawrie et al. 2021). While our understanding of the extent of this biodiversity has improved (Islam et al. 2024; Lawrie, Chaplin, Kirkendale, et al. 2023; Lawrie et al. 2025; Meusel and Schwentner 2017; Pinceel et al. 2013; Rahman, Chaplin, Lawrie, Islam, and Pinder 2025), salt lakes in the arid Australian interior remain poorly surveyed due to logistical challenges as a result of remoteness and infrequent filling regimes (Lawrie et al. 2021; Williams 2000). Opportunistic surveys during filling events may also only encompass a subset of the community composition present because species assemblages shift from a relatively diverse suite of hyposaline taxa shortly after the initial filling event into a restricted set of hypersaline species as evaporation increases (Timms et al. 2006). Regardless, given filling events are uncommon, the aquatic biodiversity of these environments is mostly surveyed and monitored using ‘rehydration trials’ where water is artificially added to collected sediment to hatch out the egg bank under controlled laboratory conditions (Campagna 2007).

Rehydration trials assume that the artificial hatching conditions provided in the laboratory will meet the hatching requirements for all taxa present and infer that the taxa observed are representative of the fauna present under natural filling conditions. However, given it is difficult to replicate natural conditions in the laboratory and specific cues for emergence for these macroinvertebrates remain poorly studied, the suite of species recovered may not necessarily be representative of the species composition present during lake inundation events (Brendonck 1996). Once taxa have emerged, they are collected, sorted, and identified to varying levels of taxonomic resolution, largely dependent on the available taxonomic expertise. This is a time‐intensive process which requires a high degree of taxonomic proficiency with identifications prone to misclassifications (Stribling et al. 2008), particularly in cryptically diverse, microscopic groups (Pfrender et al. 2010).

Bulk DNA metabarcoding of egg banks in sediments could provide a complementary method to rehydration trials for surveying aquatic macroinvertebrate biodiversity in dry salt lakes (Rahman, Chaplin, Lawrie, and Pinder 2025). DNA metabarcoding identifies multiple species from mixed DNA samples by targeting short, taxonomically informative gene regions with high‐throughput sequencing. However, the reliability and consistency of metabarcoding data are highly dependent on optimised sample collection and preprocessing protocols (Alberdi et al. 2018; Zinger et al. 2019). In particular, direct DNA extraction from unprocessed sediments often yields poor recovery of aquatic macroinvertebrates due to a combination of unspecific primer bonding, predominance of non‐target DNA, presence of inhibitors, and the limited mass of sediment used for extraction (Pawlowski et al. 2022). One strategy to improve target detection is to isolate the biological material from sediments prior to DNA extraction using density‐based separation. For example, resting macroinvertebrate eggs can be separated from sediments by mixing concentrated sucrose solutions (sugar flotation method; Onbé 1978) with sediment samples causing the less‐dense organic material (e.g., resting stages) to float out of the largely mineralised sediments (Briski et al. 2013). Sugar‐flotation has previously been used in conjunction with single species DNA barcoding (Briski et al. 2011) and for metabarcoding of the sediments from a permanent freshwater lake (Wang et al. 2020), but its efficacy for surveying aquatic macroinvertebrates in dry lakes remains undetermined.

Here we evaluate the utility of DNA bulk metabarcoding to survey the biodiversity of aquatic macroinvertebrates of dry sediments from an Australian salt lake via two complementary experiments. Experiment 1 tested whether elutriating sediments through sugar‐flotation improves the detection of aquatic macroinvertebrates compared to DNA extraction from unaltered sediment (hereafter the standard sediment method). Experiment 2 examined how sediment weight influences recovered species richness and community composition of aquatic macroinvertebrates by comparing four sugar‐floated sediment weights (50, 250, 500 and 1000 g). The hypothesis is that the observed species richness will increase with sediment weight, while the variation in community composition between replicates within a treatment will decrease. Finally, DNA metabarcoding results from the 1000 g samples from Experiment 2 were compared against taxa recovered from rehydration trials from the same sediment samples to evaluate the strengths and limitations of each method for surveying dry salt lakes.

## 2 Methods

### 2.1 Site Selection

Lake Way is a large ephemeral salt lake in central Western Australia that receives enough rainfall to fill approximately once every decade (Emerge 2020). Lake Way was selected because of its well documented macroinvertebrate fauna due to ongoing environmental monitoring targeted at assessing the impact of mining operations on the lake (Bennelongia Environmental Consultants 2020).

**FIGURE 1:**
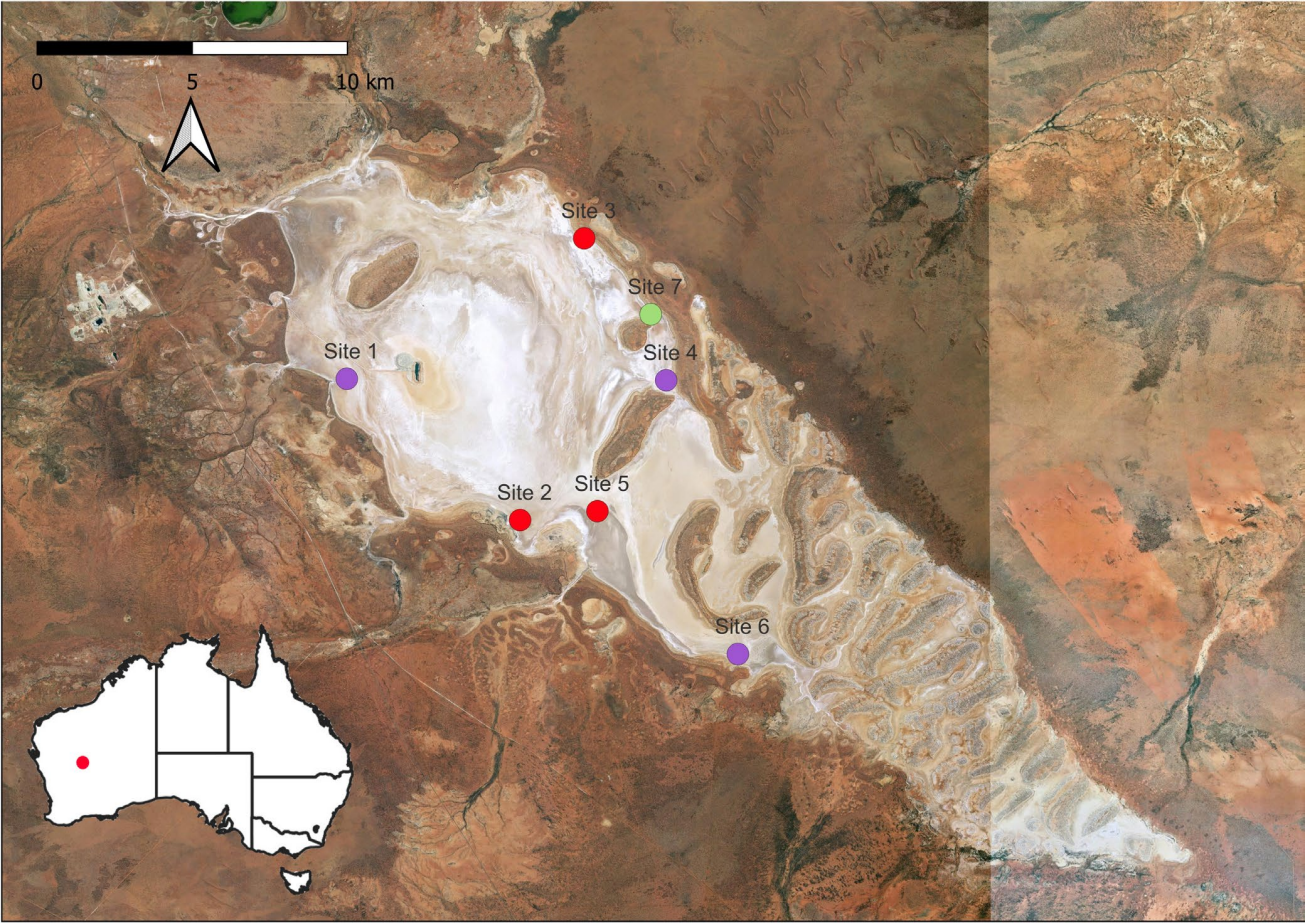
Lake Way, a large ephemeral salt lake in central Western Australia. Sites where sediment samples were collected are coloured by their inclusion in either or both of the two experiments. Purple and red dots = Experiment 1 and red and green dots = Experiment 2 sites.

Sediments for Experiment 1 were collected from six sites in November 2023, while sediment for Experiment 2 was collected from four sites in November 2024 (Table S1; Figure 1). Sediment collection involved scraping the top 10 cm of sediment in a 0.5 m^2^ area, which was stored in a calico bag and transported to the laboratory where it was homogenised via sieving (mesh size = 500 μm) and kept at room temperature for 6 months until analysis (as per Campagna 2007). Collection sites were selected based on the evidence of a previous lake highwater mark with the intention of maximising the chances of collecting dormant resting eggs (see Rahman, Chaplin, Lawrie, and Pinder 2025).

### 2.2 Experimental Design

All sample processing and extraction was undertaken in the Trace and Environmental DNA (TrEnD) laboratories, Curtin University, Western Australia, which are dedicated to working with samples of low DNA concentration.

#### 2.2.1 Experiment 1

At each of the six sites (Figure 1), a homogenised sediment sample was subdivided into six biological replicates and subjected to two different pre‐DNA extraction procedures. Three biological replicates per site were extracted following the standard method (*n* = 18) and the remaining three were subjected to the sugar‐flotation method (*n* = 18).

The standard DNA extraction method comprised processing 250 mg of sediment (the maximum input specified by the manufacturer) using the DNeasy PowerSoil Pro Kit (Qiagen) as per the manufacturer's instructions. Sugar‐flotation permits a substantially larger sediment mass to be represented in one extraction than a column‐based kit can accept directly, and therefore each biological replicate comprised 30 g of sediment in 50 mL Falcon tubes that were then topped up with 20 mL of a sucrose solution (1:1 sugar and ddH_2_O as per Onbé 1978). Falcon tubes were then hand shaken vigorously for 20 s and centrifuged at 3667 rcf for 3 min. The sucrose solution supernatant was then poured into a sterilised collection cup of a peristaltic Pall Sentino Microbiology pump (Pall Corporation), washed with ddH_2_O and filtered through a Pall 0.45 μm GN‐6 Metricel mixed cellulose ester membrane. The presence of resting eggs from ostracods, cladocerans and anostracans on the filter membrane was confirmed via microscopy in preliminary trials. The filter membrane was then processed using the DNeasy PowerSoil Pro Kit as per the manufacturer's instructions. All materials were decontaminated between samples using a 10% bleach solution, and two blank extraction controls (reagents only) were processed through to sequencing to assess potential cross contamination between samples. Extractions were undertaken in a dedicated PCR‐free laboratory. The metabarcoding and bioinformatics methods were the same as presented in Experiment 2 (see below).

#### 2.2.2 Experiment 2

Additional sediment samples were collected in November 2024 from three sites sampled for Experiment 1 (Site 2, Site 3 and Site 5) and an additional site (Site 7). Site selection for Experiment 2 was constrained by a partial flooding event in 2024 with the selected sites representing unflooded areas. Sediment from each site was added into pre‐decontaminated plastic containers (10% bleach) in four weight classes (50, 250, 500 and 1000 g) with three replicates per weight class. A 1:1.5 ratio of sediment to sucrose solution (1:1 sugar to ddH_2_O as above) was added to each sample container and mixed thoroughly with a decontaminated stirring stick for approximately 1 min per sample. Large sample volumes prevented centrifugation as per Experiment 1 but to minimise filter paper clogging with fine suspended solids, samples were left to settle for approximately 4 h prior to filtering. After settling, the sucrose solution was filtered through a 63 μm sieve and washed with ddH_2_O into a sterile collection vessel and filtered through a Pall 0.45 μm GN‐6 Metricel mixed cellulose ester membrane (47 mm) using a peristaltic Pall Sentino Microbiology pump (Pall Corporation). The filter paper was then added to a DNeasy PowerSoil Pro Kit zirconium bead tube and processed as per the manufacturer's instructions. Extractions were undertaken in a dedicated PCR‐free laboratory, and with two DNA extraction controls that were processed alongside the samples through to sequencing.

### 2.3 Metabarcoding

The DNA extracts from both experiments were quantified using a Qubit 4.0 fluorometer (Life Technologies) and then qPCR amplified using the fwhF2/fwhR2n primer set (F: 5′‐GGDACWGGWTGAACWGTWTAYCCHCC‐3′; R: 5′‐GTRATWGCHCCDGCTARWACWGG‐3′), hereafter referred to as fwh, designed for the amplification of approximately ~205 bp of the *cytochrome oxidase subunit 1* (COI) (Vamos et al. 2017) and Crust16S primer set (F: 5′ GGGACGATAAGACCCTATA 3′; R: 5′ ATTACGCTGTTATCCCTAAAG 3′), designed for the amplification of approximately ~170 bp of 16S (Berry et al. 2017). These assays were selected because they have been designed for the amplification of aquatic macroinvertebrates (fwh) including crustaceans (Crust16S). Prior to metabarcoding, the optimal DNA dilution level (neat, 1:10 or 1:100 dilution) for each sample was determined via qPCR. The qPCRs consisted of a 25 μL reaction comprised of: 1× PCR Gold Buffer (Applied Biosystems), 0.25 mM dNTP mix (Astral Scientific, Australia), 2 mM MgCl_2_ (Applied Biosystems), 1 U AmpliTaq Gold DNA polymerase (Applied Biosystems), 0.4 mg/mL bovine serum albumin (Fisher Biotec), 0.4 μM forward and reverse primers, 0.6 μL of a 1:10,000 solution of SYBR Green dye (Life Technologies), 2 μL template DNA and PCR H_2_O up to 25 μL. qPCRs were performed on StepOne Plus instruments (Applied Biosystems) with the following cycling conditions for fwh: 95°C for 5 min, followed by 50 cycles of: 95°C for 30 s, 50°C for 30 s, 72°C for 45 s, then a melt‐curve analysis of: 95°C for 15 s, 60°C for 1 min, 95°C for 15 s, finishing with a final extension stage at 72°C for 10 min. The cycling conditions for Crust16S were the same except that the annealing temperature was 51°C.

The DNA metabarcoding was performed using a one‐step qPCR with fusion primers and the same reagents and cycling conditions as described above but all fusion‐tagged qPCR reactions were prepared in dedicated clean room facilities using an automated QIAgility robotics platform (Qiagen). Fusion tagged qPCRs included at least one non‐template control (reagents only; NTC) and a positive control (Libellulidae, *Tramea limbata*) included in every run with controls processed alongside samples through to sequencing for both fwh (extraction controls = 4, NTC = 3, positive controls = 3) and Crust16S (extraction controls = 4, NTC = 2, positive controls = 1). Each biological replicate was repeated in two PCR technical replicates to account for detection sensitivity. Each technical replicate contained a unique combination of two 8 bp multiplex identifier‐tags (MID‐tag), one on each of the forward and reverse primers as described in Koziol et al. (2019) and van der Heyde et al. (2020).

Sequencing libraries were prepared separately for the fwh and Crust16S assays, by combining samples into mini‐pools based on the qPCR amplification results from each sample. Samples were assigned to mini‐pools according to comparable ΔRn fluorescence endpoints, with ΔRn values compared only among samples amplified within the same qPCR run. The mini‐pools were then quantified for DNA concentration (ng/μL) using a QIAxcel fragment analyser (Qiagen) and combined at approximate equimolar concentrations to form the sequencing libraries. Amplicons in each library were then size selected to 150–400 bp (Pippin Prep: Sage Sciences) using a 2% Agarose Gel Cassette (Sage Science), cleaned using a QIAquick PCR purification kit (Qiagen), quantified by Qubit 4.0 Fluorometer (Thermo Fisher), and diluted to 2 nM. All libraries were sequenced on an Illumina NextSeq 2000 sequencing platform following the manufacturers protocols, utilising a NextSeq1000/2000 P1 Reagents 300 cycle kits, with a final library molarity of 650 pM containing 24% PhiX.

### 2.4 Bioinformatics

Sequence data was processed using the automated workflow ‘eDNAFlow’ (Mousavi‐Derazmahalleh et al. 2021) on the Setonix cluster at the Pawsey Supercomputing Centre (Pawsey Supercomputing Research Centre 2023), with parameters ‐‐maxTarSeq '10', ‐‐minLen '100', ‐‐minsize '8', ‐‐qcov '100', ‐‐perc_identity '85' for COI and '90' for 16S, and ‐‐minMatch_lulu '97'. The ZOTUs (zero‐radius operational taxonomic units) were compared using BLASTN against the NCBI (GenBank) and an in‐house custom reference database generated from known taxa collected from previous rehydration trials and active sampling of the seven studied sites (see Supplementary Table 2 for GenBank accession numbers). A minimum pairwise identity threshold of 85% was applied for COI and 90% for 16S. When two or more taxa matched a ZOTU and their percentage identities differed by ≤ 0.5%, the assignment was reduced to the lowest taxonomic rank shared by those taxa. All taxonomic assignments were assessed manually, and species‐level attributions were limited to matches > 92% for COI and > 97% for 16S.

The curated ZOTU table from *lulu* (Frøslev et al. 2017) was filtered using a multi‐step decontamination process using *metabar* (Zinger et al. 2021) in R Studio (R Core Team 2024). ZOTUs that were most abundant in at least one control were considered contaminants and removed using the contaslayer function, while ZOTUs occurring at a relative abundance below 0.5% within an individual PCR technical replicate were considered potential tag‐jumps and removed with the tagjumpslayer. PCR technical replicates were aggregated by biological replicate with taxa in at least one biological replicate at a site recorded as a positive detection.

Bioinformatic outputs were manually verified and automated taxonomic assignments refined for each primer dataset as follows:

1. Non‐arthropod sequences were removed (e.g., rotifer, fungal, or diatom ZOTUs).
2. ZOTUs assigned to terrestrial arthropod species were removed (e.g., Arachnida or Blattodea).
3. Filtered ZOTUs sequences of uncommon, broadly assigned or not previously recorded taxa at a site were manually verified and reassigned accordingly.

### 2.5 Rehydration Trials

The same sediment samples from the four sites in Experiment 2 (Site 2, Site 3, Site 5 and Site 7) were used in the rehydration trials following the methods of Campagna (2007). Specifically, 500 g of sediment was placed into a 20 L glass aquarium with approximately 18 L of reverse‐osmosis water, which was aerated using an airstone and maintained under a 12‐h light/dark cycle in a temperature‐controlled room set to 25°C. Temperature, electrical conductivity, pH and percentage saturation of dissolved oxygen were recorded three times per week using a multiparameter water quality meter (Table S7). Aquaria were not topped up with water after the initial flooding event with water allowed to gradually evaporate over the course of the experiment. Aquaria were monitored for 28 days; then any remaining water was removed, and the sediments were dried using heat lamps. Dry sediments were subjected to a second filling cycle. This process is necessary to assess for the presence of ‘bet hedging’ taxa within the egg bank which may not respond to the initial filling event (Pinceel et al. 2021). All emergent animals were harvested at the end of each filling cycle by pouring the water from each aquarium through a 53 μm sieve. All harvested taxa were identified to the lowest possible taxonomic level.

### 2.6 Data Analysis

Data for each assay were pooled into separate presence/absence datasets per experiment with species detected by both assays counted only once. To examine how sediment weight influenced the detection of taxa in Experiment 2, species richness was analysed using linear mixed‐effects models in R Studio (R Core Team 2024) using the lme4 package (Bates et al. 2015). Sediment weight was first modelled as a continuous variable to test for an overall linear relationship between sediment weight and species richness, while including site as a random intercept to account for non‐independence among samples from the same location. Because sediment weights represented discrete treatment levels (50, 250, 500 and 1000 g), a second model was fitted with sediment weight as a categorical factor to enable post hoc pairwise comparisons among weight levels. Tukey‐adjusted comparisons of estimated marginal means were performed using the *emmeans* package. Model assumptions were assessed by inspection of residual against fitted plots and homogeneity of residual variance among weight classes was tested using Levene's test. No violations of model assumptions were detected. The effect of sediment weight, site, and their interaction on community composition was analysed using permutational multivariate analysis of variance (PERMANOVA) based on Bray‐Curtis dissimilarities (999 permutations) using the *vegan* package (Dixon 2003).

To test whether increasing sediment weight reduced among‐replicate variability in community composition within each weight class, we quantified multivariate dispersion using PERMDISP (betadisper function in the *vegan* package). Bray‐Curtis dissimilarities were calculated from presence‐absence community matrices. Because sites differed strongly in community composition (see below), PERMDISP was run separately for each site, with sediment weight (50, 250, 500 1000 g) as the grouping factor. This approach was taken to ensure that differences reflected variability between weight classes rather than inherent community differences among sites.

For the comparison between metabarcoding and rehydration trials, we limited the metabarcoding data to the 1000 g treatment of Experiment 2 with replicates pooled to the site level. This treatment was selected because it was the most diverse of the four sediment treatments analysed (see Section 3). To account for differences in taxonomic resolution between the metabarcoding and the morphological analyses, all species‐level identifications were brought up to genus prior to comparison. A Kruskal–Wallis test was used to determine whether median taxa richness differed significantly between the two sampling methods.

## 3 Results

The two DNA metabarcoding assays (fwh and Crust16S) generated a combined total of 81.3 million reads across both experiments. For Experiment 1, the fwh assay produced 10.1 million raw reads, of which 2.7 million reads and 23 ZOTUs remained after removal of unassigned, contaminant, and non‐target sequences (Table S3). The Crust16S assay yielded 8.2 million raw reads, resulting in 7.4 million reads and eight ZOTUs retained after all filtering stages (Table S3). For Experiment 2, the fwh assay produced 37 million reads, which, following decontamination and removal of non‐target sequences, was reduced to 14.5 million reads and 151 ZOTUs (Table S3). The Crust16S assay generated 25.9 million raw reads, with 23.4 million reads and 43 ZOTUs retained post‐filtering (Table S3).

### 3.1 Experiment 1: Unaltered Sediment vs. Sugar‐Flotation

Using the fwh assay, the final dataset comprised 17 samples with an average of 152,138 (SE ± 35,411) reads per sample and a total of 23 ZOTUs. For the Crust16S library, the final dataset comprised nine samples with an average read depth of 827,550 (SE ± 235,282) reads and a total of eight ZOTUs. Essentially all samples prepared with the sugar‐flotation method identified at least one ZOTU from one of the two assays (fwh = 17/18, Crust16S = 9/18 samples). Except for a single sample from the fwh dataset, samples extracted using the standard sediment DNA extraction method failed to detect any target ZOTUs (i.e., those assigned to aquatic macroinvertebrates).

The 23 ZOTUs detected in the fwh dataset were assigned to five aquatic macroinvertebrate species, while the eight ZOTUs from the Crust16S assay matched five taxa. Only crustacean species were recorded, with *Parartemia laticaudata* and *Triops* sp. BV‐2012 detected by both fwh and Crust16S, while the remaining six species were detected by a single assay (Table S4).

**FIGURE 2:**
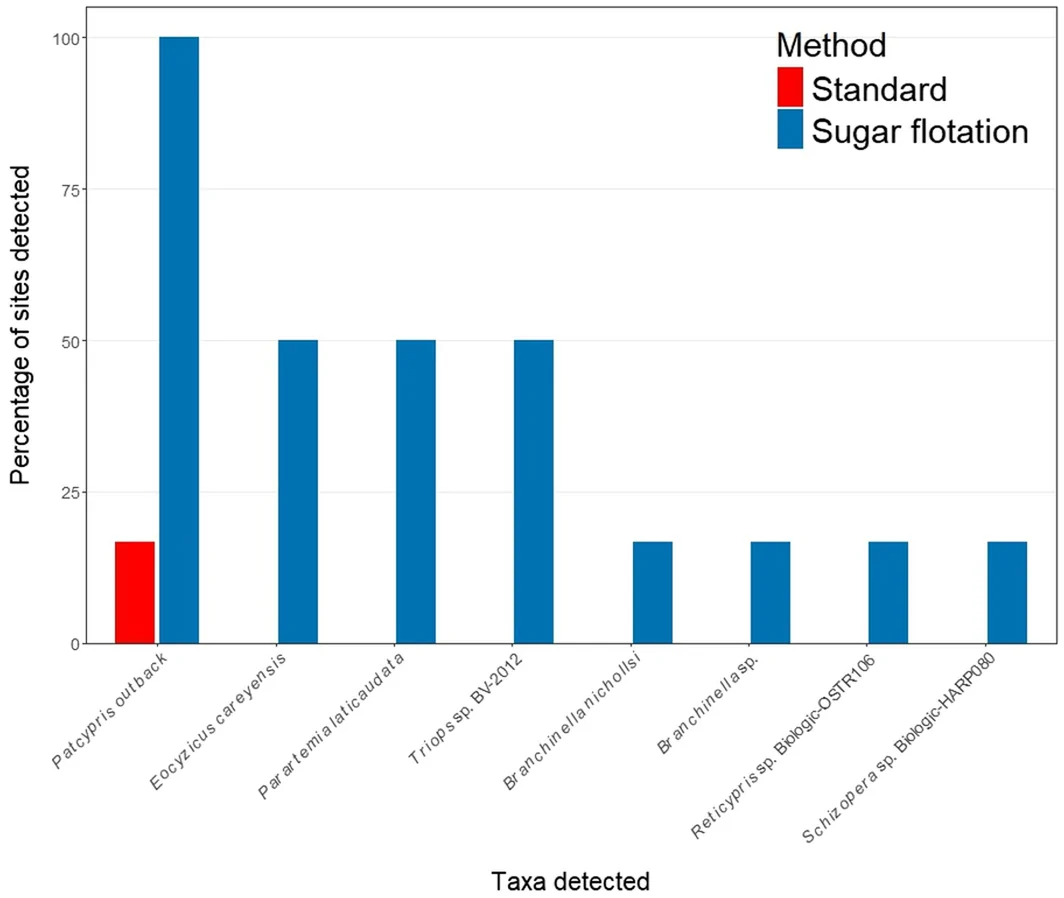
Percentage of sites in which each taxon was detected using metabarcoding following standard sediment DNA extraction (red) and sugar‐flotation (blue) treatments. Detection frequencies are shown for each taxon as the percentage of the six sites that had at least one positive detection, with results from both metabarcoding assays combined into a single presence–absence record per taxon.

Using the combined fwh and Crust16S dataset, the sugar‐flotation method significantly outperformed the standard method. All taxa were detected exclusively using the sugar‐flotation method except for a single *Patcypris outback* detection in one standard sample (Figure 2; Table S4). *Patcypris outback* was detected in all sites, and *Triops* sp. BV‐2012, *Parartemia laticaudata* , and *Eocyzicus careyensis* were also common. The detections of the remaining species were sporadic, with four taxa (*Reticypris* ‘sp. Biologic‐OSTR106’, *Branchinella nichollsi* , *Branchinella* sp., and *Schizopera* ‘sp. Biologic‐HARP080’) only identified from one site (Table S4).

### 3.2 Experiment 2: Sediment Mass for Sugar‐Flotation

Of the 48 samples sequenced, 45 fwh samples (mean read depth = 323,289 ± 22,547 SE) and 35 Crust16S samples (mean read depth = 668,332 ± 72,283 SE) passed all bioinformatic and quality filtering criteria (Table S3). The breakdown of reads per sediment‐weight category for each assay is in Table S5.

**FIGURE 3:**
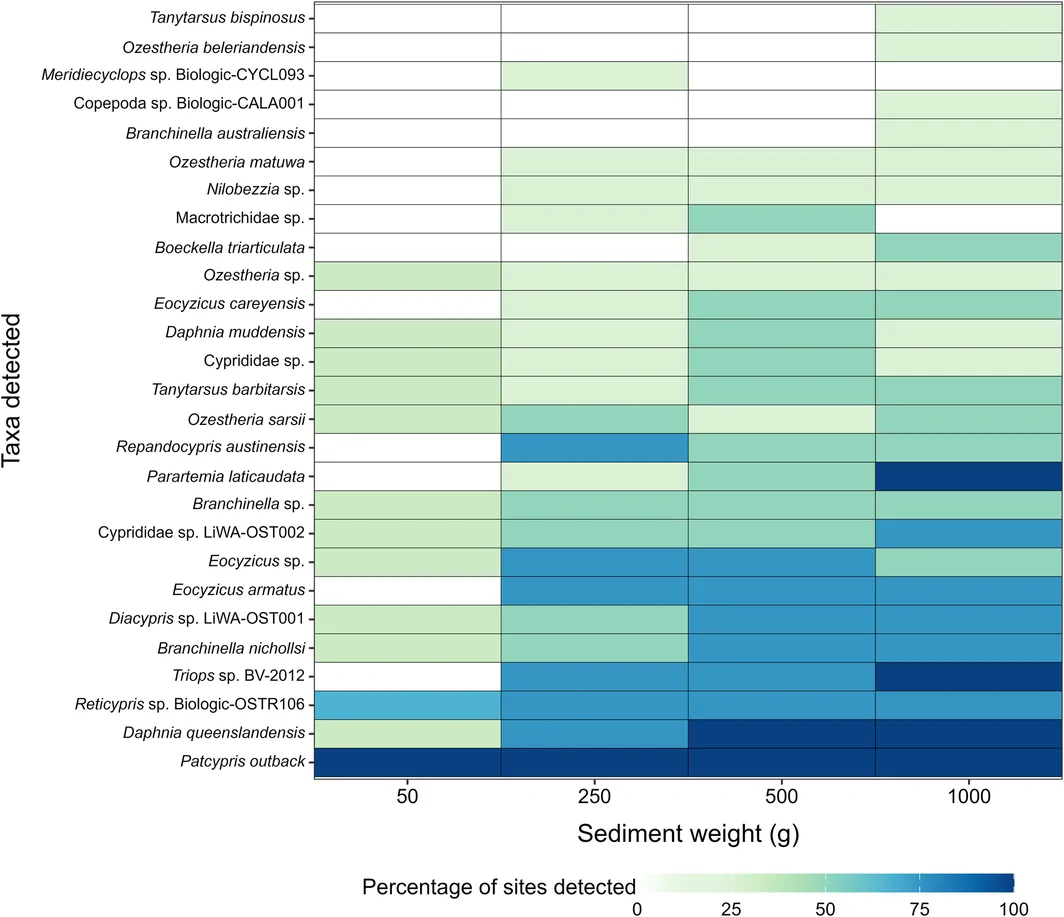
Taxa detected across the four sugar‐floated sediment weight categories used in this study. Colour of the square reflects the percentage of sites where the taxa were detected.

Across all samples, the fwh assay detected 151 ZOTUs assigned to 24 aquatic macroinvertebrate taxa, while the Crust16S assay yielded 43 ZOTUs assigned to 11 taxa (Table S6). Across both assays, a total of 27 taxa were detected, comprising 16 taxa unique to fwh, three unique to Crust16S and eight taxa shared between the two assays. Similar to Experiment 1, most species detected were crustaceans (*n* = 24), although three dipterans from the Ceratopogonidae and Chironomidae were also identified. Diplostraca (seven taxa) and Ostracoda (six taxa) were the two most speciose groups, with Anostraca (four taxa), Cladocera (three taxa), Calanoida (two taxa), Cyclopoida (one taxon) and Notostraca (one taxon) also detected. *Branchinella nichollsi* , *Daphnia queenslandensis*, *Eocyzicus armatus*, *Eocyzicus* sp., *Parartemia laticaudata* , *Patcypris outback* and *Triops* sp. BV‐2012 were detected in all sites at least once, while *Branchinella australiensis* , Copepoda ‘sp. Biologic‐CALA001’, *Meridiecyclops* ‘sp. Biologic‐CYCL093*’, Ozestheria beleriandensis*, *O. matuwa*, *Ozestheria* sp., *Tanytarsus bispinosus* and *Nilobezzia* sp. were detected in a single site (Figure 3).

**FIGURE 4:**
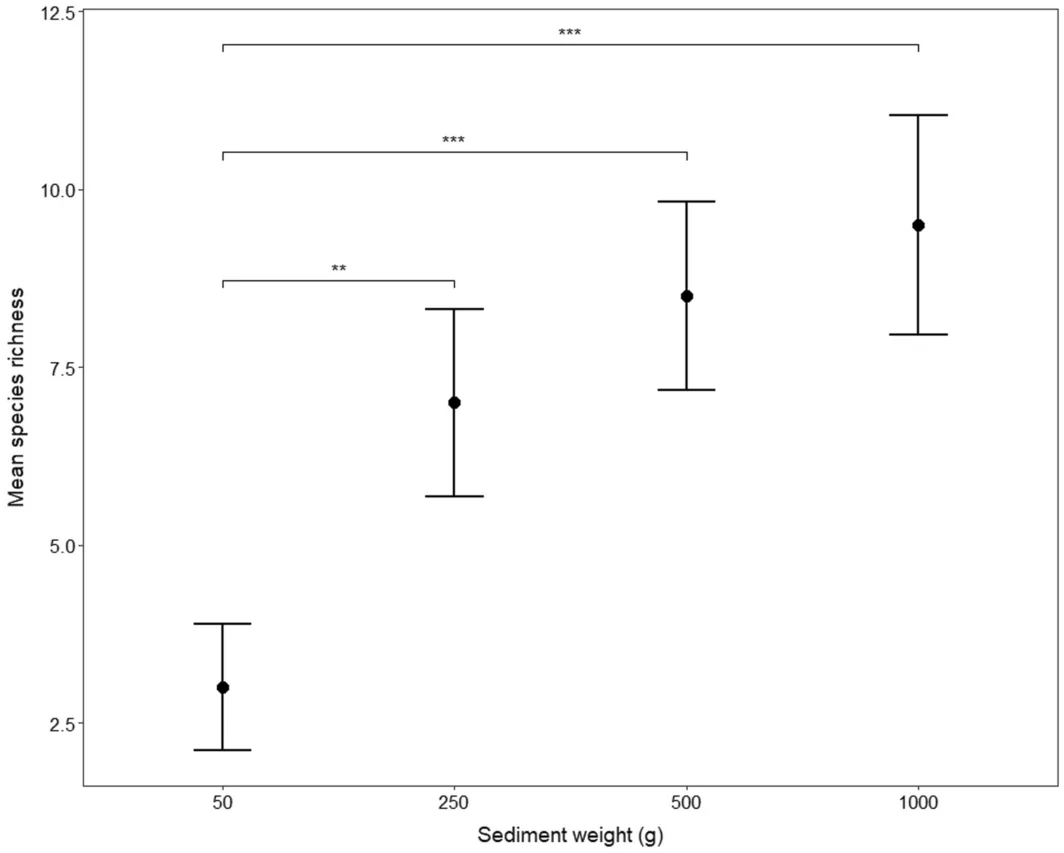
Dot plot displaying the mean (± standard error) species richness detected from increasing sediment weights (50, 250, 500, and 1000 g) following sugar‐flotation. Bars and asterisks between dots represent statistical significance (**p < 0.01, *** = *p* < 0.001). Data from fwh and Crust16S have been pooled with co‐detected species recorded only once.

Using the combined dataset of both assays, the 250 g (*n* species = 22), 500 g (*n* species = 22) and 1000 g (*n* species = 25) sediment treatments detected essentially the same total number of species and were all higher than the 50 g treatment (*n* species = 13; Figure 3). Average species richness increased significantly with sediment weight (LMM; *F*_1,43_ = 29.7, *p* < 0.001), showing a strong positive linear trend (*t*_43_ = 5.08, *p* < 0.001). After accounting for site‐level variation, mean species richness increased from an average of 3 ± 0.8 species at 50 g to 9.5 ± 1.5 species per sample at 1000 g of sediment (Figure 4). Post hoc Tukey tests revealed that 50 g samples contained significantly fewer species than 250 g (*p* = 0.001), 500 g (*p* < 0.001), and 1000 g (*p* < 0.001) treatments, whereas differences among the higher sediment weights were not significant.

**FIGURE 5:**
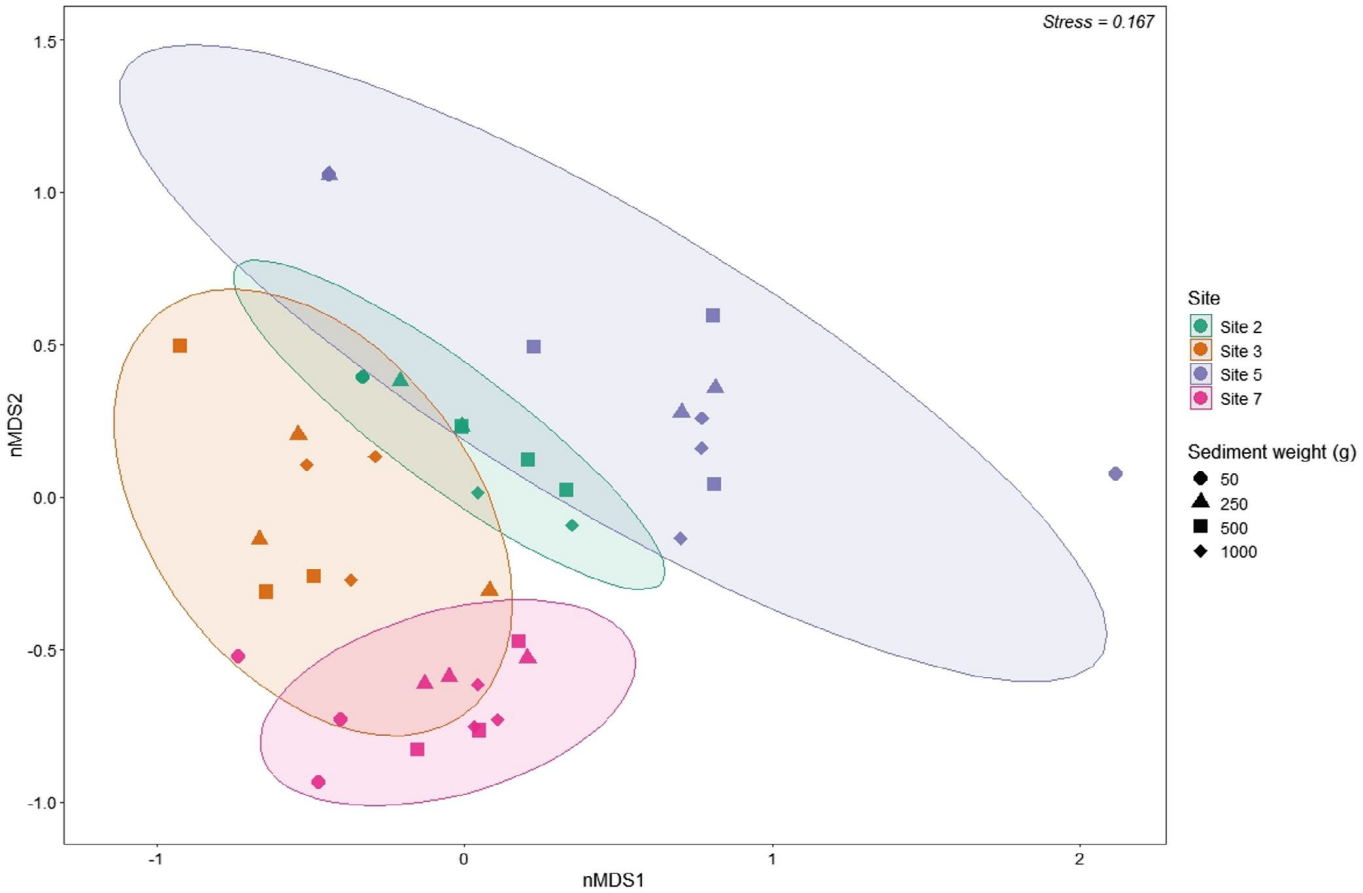
nMDS plot displaying community compositional differences between sites (colours) and sediment levels (shapes) in metabarcoding data generated from Lake Way sediment samples. Shaded ellipses represent 95% confidence regions for each site.

Community composition analyses based on Bray–Curtis dissimilarities revealed significant effects of sediment weight (PERMANOVA; *F*_3,29_ = 2.37, *R* ^2^ = 0.08, *p* = 0.008) and site (*F*_3,29_ = 15.94, *R* ^2^ = 0.51, *p* = 0.001). The interaction between sediment weight and site was not significant (*F*_8,29_ = 1.1, *R* ^2^ = 0.09, *p* = 0.32). Differences between sites explained a substantially greater proportion of the variation in community composition (51%) than different sediment weights (7.6%), indicating that differences among sites were the dominant driver of community structure. This trend is reflected in Figure 5 where samples from the same site tended to cluster together with less separation between the different sediment categories within each site.

**FIGURE 6:**
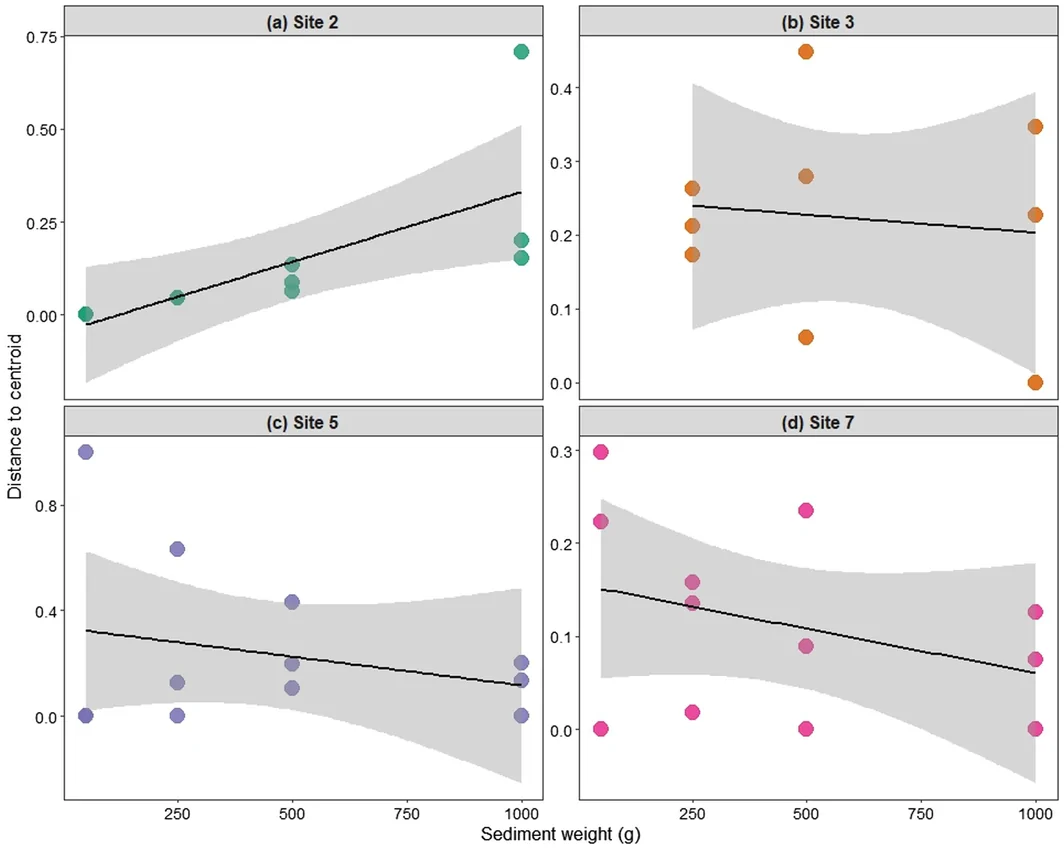
Site‐level patterns of community dispersion (Bray‐Curtis distance to group centroid) across sediment weight classes. Each panel shows a separate site: (a) Site 2, (b) Site 3, (c) Site 5, and (d) Site 7. Points represent individual replicate samples, and lines show linear regressions with 95% confidence intervals.

We detected no reduction in the variability of community composition among replicate samples within weight classes as sediment weight increased. PERMDISP analyses showed no significant differences in within‐group dispersion for any site (permutation *p*: Site 7 = 0.2; Site 2 = 0.3; Site 3 = 0.3; Site 5 = 0.9). Three sites (e.g., Site 7, Site 3 and Site 5) showed non‐significant trends of declining variance with increasing sediment weight while one site (e.g., Site 2) displayed the reverse (Figure 6).

### 3.3 Method Comparison: Metabarcoding vs. Sediment Rehydration

**FIGURE 7:**
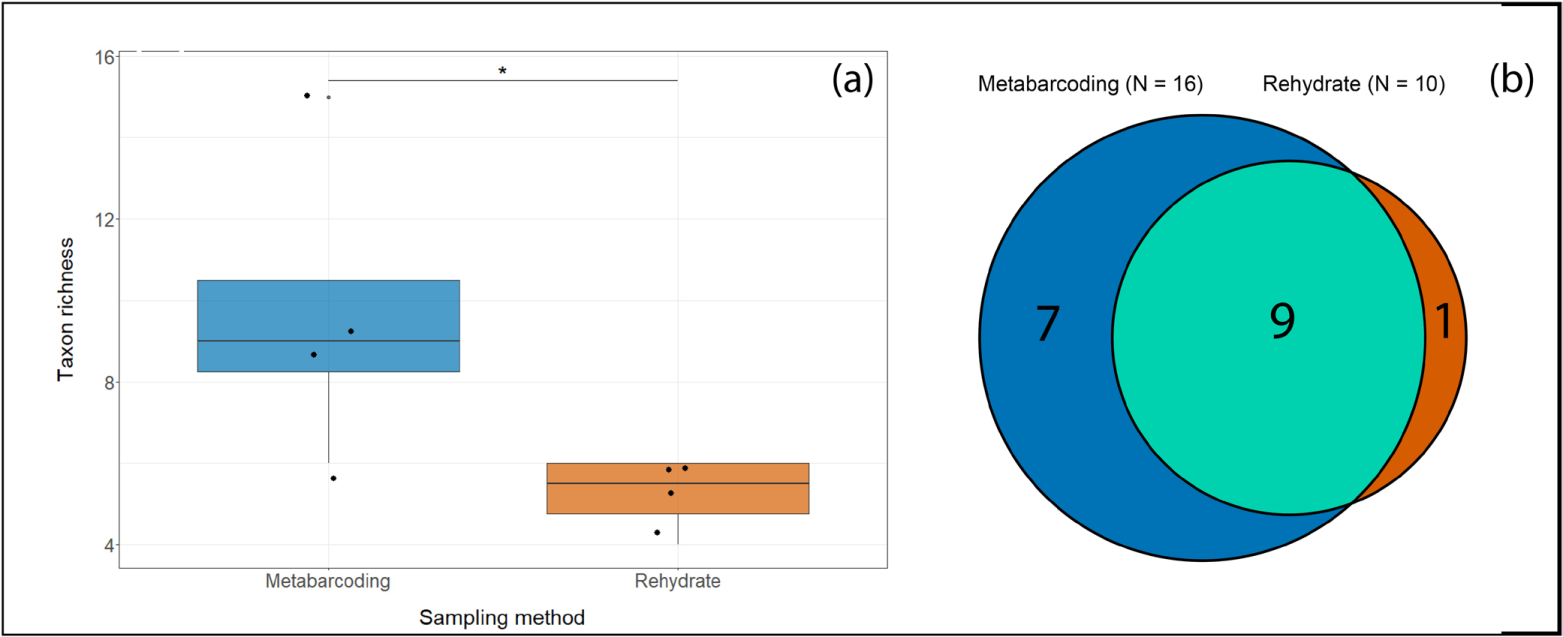
(a) Boxplot showing the difference in the number of taxa identified between the metabarcoding (blue) and rehydration trials (orange). Metabarcoding data are from the 1000 g treatment in Experiment 2. * indicates that difference in taxon richness between the two methods was statistically significant (*p* = 0.037). (b) Venn diagram comparing the taxa identified via only metabarcoding (blue) and rehydration trials (orange) and taxa identified by both methods (green) from dry sediments collected from Lake Way. *N* = total number of taxa identified by either method. To account for differences in taxonomic resolution between the two methods, all species level identifications have been raised to genus.

The metabarcoding approach (median = 9.5 taxa, range = 8–15) identified significantly more taxa (*χ* ^2^ = 4.3, *p* = 0.037) than the rehydration trials (median = 5.5 taxa, range = 4–6; Figure 7a). The two methods shared most taxa (9 of 16), though the metabarcoding detected more unique taxa (7 unique taxa) than the rehydration trials (1 unique taxon; Figure 7b). Two dipterans (*Tanytarsus* and *Nilobezzia*) and one ostracod (Cyprididae), cladoceran (Macrothricidae), calanoid (*Boeckella*), anostracan (*Branchinella*), and Spinicaudata (*Ozestheria*) were detected in the metabarcoding data but not present in the rehydration trials, while one cladoceran (*Macrothrix*) hatched but was not identified by metabarcoding.

## 4 Discussion

This study demonstrates that sugar‐flotation combined with metabarcoding is an effective approach for recovering aquatic invertebrate biodiversity data from dry lake sediments in ephemerally inundated environments and records more taxa than conventional methods (rehydration trials). We also showed that increasing the initial mass of sediment for the sugar‐flotation treatment significantly increases the number of aquatic macroinvertebrate species recovered, although there are diminishing returns at higher sediment weights.

### 4.1 Benefits of Combining Sugar‐Flotation and DNA Metabarcoding

In Experiment 1, the sugar‐flotation method consistently detected aquatic invertebrate taxa, with nearly all sugar‐floated samples detecting at least one target taxon, whereas only a single standard‐extraction sample resulted in a positive detection. The results of the standard extraction method are consistent with previous studies which have found that unprocessed sediment samples are an inefficient method of detecting macroinvertebrates (Pawlowski et al. 2022). Two features of the sugar‐flotation workflow likely contribute to this difference. The first is the composition of the material extracted. Although DNA in sediments can be both extracellular and intracellular (Ellegaard et al. 2020), the significant difference in the performance of the sugar‐floated versus standard samples is likely in part due to the differential efficiency in recovering DNA from macroinvertebrate resting stages (although other sources of DNA cannot be excluded, see below). Elutriation increases the proportion of aquatic macroinvertebrate DNA recovered from a sample and reduces interference from non‐target DNA sources in the sediment matrix (Macher et al. 2018), which can otherwise dominate the DNA pool and obscure detection of rare taxa. The second is the mass of sediment each workflow can process, which is a property of the methods themselves rather than an incidental difference between treatments. The column‐based kit used here accepts a maximum of 250 mg of sediment, and even large input formats designed for high biomass samples accept no more than approximately 10–15 g (e.g., DNeasy PowerMax Soil Kit, Qiagen) while the sugar‐flotation is less restricted by larger sediment fractions (see below).

### 4.2 Effect of Sediment Weight on Taxa Detection

As expected, species richness increased with the amount of sediment treated with sugar‐floatation. This increase was most notable from 50 to 250 g with no significant difference in species richness among the 250, 500 and 1000 g weight classes, although the 1000 g samples did detect the most taxa overall. These findings are consistent with previous studies using metabarcoding on sediment samples from non‐lake environments targeting metazoans and non‐metazoan eukaryotes (Nascimento et al. 2018), bacteria (Ellingsøe and Johnsen 2002), fungi (Penton et al. 2016) and soil macroinvertebrates (Kirse et al. 2021), which found that increasing the amount of source material for DNA extraction also provides a more comprehensive assessment of the biodiversity present. However, increasing sediment weight within a site was not correlated with a significant reduction in within‐treatment variation, though some sites did show a trend toward increasing the similarity of the communities recovered by replicates at greater sediment weight. This was contrary to our expectations that within a site larger sediment treatments would have lower variation in the community composition recovered between replicates because processing more sediment would capture more of the inherent spatial variability of resting eggs within sediments (Rahman, Chaplin, Lawrie, and Pinder 2025). This may reflect insufficient biological replication to detect this effect, suggesting that increased sediment weight alone is unlikely to compensate for limited sampling effort when characterising spatially heterogeneous egg banks. Pooling separate DNA extracts from a site prior to PCR has been shown to reduce within‐site heterogeneity in marine sediments (Hestetun et al. 2021) and could be potentially used to improve the consistency of the results generated here. Together our results suggest that metabarcoding biodiversity surveys of aquatic macroinvertebrates in dry lake sediments should sugar‐float 1000 g of sediment where logistically feasible. The 1000 g treatment recovered both the highest mean species richness and the greatest total number of taxa across sites, and although it could not be statistically separated from the 250 and 500 g treatments, there is no indication that returns had ceased by 1000 g. Where processing capacity is limited, 250 g represents a defensible minimum, since this was the mass above which mean richness no longer increased significantly and below which detection declined markedly. Additional biological replicates should be prioritised alongside sediment mass, given that within‐site variation was not detectably reduced by increasing sediment weight alone.

### 4.3 Metabarcoding vs. Rehydration Trials

Biodiversity assessments conducted using a combination of approaches usually provide a more comprehensive depiction of biotic communities by capitalising on the complementary strengths of different methods (Schenekar 2023). A combination of different sampling approaches is considered best practice for environmental monitoring in salt lakes (e.g., Campbell et al. 2023; Saccò et al. 2025) and freshwater ecosystems more broadly (Schenekar 2023). While ultimately, we suggest that conducting both rehydration trials and DNA metabarcoding on dry lake sediments produces highly complementary information, in practice environmental managers typically face resource constraints that force pragmatic trade‐offs to select method(s) that best align with their specific objectives. The evaluation of the performance and assumptions of DNA metabarcoding and rehydration trials below has been developed within this context.

Baseline surveys of biodiversity are a key component in conservation planning that provide foundational ecological context and identify the presence of rare or threatened taxa. Consistent with previous comparisons between DNA metabarcoding and conventional biodiversity survey methods (Iacaruso et al. 2025), DNA metabarcoding detected a greater overall number of taxa, including seven unique taxa, whereas only a single taxon was exclusively recovered through rehydration trials. These findings are not surprising, given that DNA metabarcoding approaches generally have a superior ability to detect rare species and identify taxa to lower taxonomic levels compared with morphological analyses (Andersson et al. 2023; Bush et al. 2019), especially in cryptically diverse macroinvertebrate groups (Pfrender et al. 2010). The disparity between the taxa recovered from the same sediment samples between both methods may indicate that the artificial conditions used in the rehydration trials do not meet the hatching requirements of some taxa. For example, *Branchinella* was not recorded in the rehydration trials despite producing resting eggs (Timms and Lindsay 2011), being consistently detected by DNA metabarcoding, and being present in sweep net samples collected from Lake Way during inundation (Bennelongia Environmental Consultants 2020). While the absence of viable eggs cannot be ruled out as an explanation for the non‐hatching of *Branchinella* and other taxa (see below), their detections demonstrate the utility of DNA metabarcoding in establishing baseline biodiversity information for dry lakes given our currently poor understanding of the ecological requirements of many salt lake invertebrates (Lawrie et al. 2021; Lawrie, Chaplin, Rahman, et al. 2023).

It is important to note that neither rehydration trials nor metabarcoding recorded all taxa known to occur in Lake Way during inundation (Bennelongia Environmental Consultants 2020), highlighting the importance of opportunistic sampling of salt lakes during flooding conditions. Missing taxa were mostly transient insects that do not deposit resting stages in dry lakes but re‐colonise during filling events and therefore will not hatch in rehydration trials and are unlikely to be detected in the metabarcoding of lake sediments. However, calanoid, harpacticoid, and cyclopoid copepods were detected either inconsistently or not at all across the metabarcoding data, despite being abundant under flooding conditions (Biologic unpublished data) and cladocerans were inconsistently detected but common in the rehydration trials. Although a local DNA reference library was generated from previous aquatic macroinvertebrate collections at Lake Way, which included representative harpacticoid, calanoid, cyclopoid, and cladoceran species collected from Lake Way, it is possible that their non‐detection may reflect gaps in the DNA reference library used here. In addition, we used multiple DNA assays as is typically recommended in metabarcoding studies to overcome primer biases and to compensate for phylogenetically diverse environments (Corse et al. 2019; Hajibabaei et al. 2019; Zhang et al. 2018). These assays did collectively increase the taxonomic coverage of this study; however, the inconsistent detection of copepods and cladocerans suggests that the metabarcoding results could have been improved with the inclusion of additional primer sets (see Bylemans et al. 2025).

As metabarcoding data cannot differentiate between DNA from living versus dead organisms (Pawlowski et al. 2022), a detection of a species in the metabarcoding data cannot necessarily be used as evidence of viable resting stages. For example, *Tanytarsus barbitarsis* was detected via metabarcoding in multiple sites and occurs in Lake Way under flooding conditions (Biologic unpublished data) but was not present in the rehydration trials. Although *T. barbitarsis* does deposit eggs with some desiccation resistant capabilities, experimental results of Kokkinn and Williams (1988) suggest that egg viability is limited to 5 days at 20°C. However, *T. barbitarsis* can generate very high population densities within salt lakes (e.g., 140,000 individuals/m^2^; Paterson and Walker 1974) suggesting that the metabarcoding detection of *T. barbitarsis* was most likely from residual DNA deposited in the sediment during previous flooding events. Therefore, rehydration trials remain a necessary component in the monitoring programmes of ephemeral aquatic environments where the viability of resting egg banks needs to be established.

## 5 Conclusion

This study is one of few that have explored metabarcoding as a tool for surveying macroinvertebrate biodiversity in salt lake environments (Campbell et al. 2023; Saccò et al. 2025). It demonstrates that combining sugar‐flotation of dry lake sediments with DNA metabarcoding is an effective method for documenting macroinvertebrate biodiversity of ephemeral salt lakes. Although this study focused on one biotic community (aquatic macroinvertebrates) in a single lake with a limited number of sites and samples per site, the approach used here could be used to conduct biodiversity surveys of temporary lakes more broadly and focus on other organisms which produce resting stages or seed banks, e.g., rotifers or macrophytes. Utilising sugar‐flotation is critical, with our results clearly demonstrating that the sediment extraction protocol utilised here was ineffective for detecting aquatic macroinvertebrates. Increasing the initial mass of sediment had a substantial effect on the number and consistency of taxa detected, with sugar‐flotation of 1000 g recommended where feasible to detect as many aquatic macroinvertebrate taxa as possible within a site, and 250 g representing a practical minimum.

## Author Contributions

**Angus D'Arcy Lawrie:** conceptualization (lead), data curation (lead), formal analysis (lead), investigation (lead), methodology (lead), project administration (lead), writing – original draft (lead), writing – review and editing (lead). **Pia Dethlefsen:** data curation (equal), formal analysis (equal), investigation (equal). **Mahabubur Rahman:** data curation (equal), investigation (equal), methodology (equal), writing – original draft (equal), writing – review and editing (equal). **Christopher Hofmeester:** conceptualization (equal), methodology (equal), project administration (equal), writing – original draft (equal), writing – review and editing (equal). **Jessica Delaney:** methodology (equal), project administration (equal), writing – original draft (equal), writing – review and editing (equal). **Matthew A. Campbell:** formal analysis (equal), writing – original draft (equal), writing – review and editing (equal). **Joshua P. Newton:** investigation (equal), methodology (equal), writing – original draft (equal), writing – review and editing (equal). **Marina Elisa de Oliveira:** investigation (equal), methodology (equal), writing – original draft (equal), writing – review and editing (equal). **Joel Huey:** conceptualization (equal), methodology (equal), project administration (equal), writing – original draft (equal), writing – review and editing (equal). **Morten E. Allentoft:** investigation (equal), methodology (equal), supervision (equal). **Mattia Saccò:** investigation (equal), methodology (equal), supervision (equal), writing – original draft (equal), writing – review and editing (equal).

## Funding

Funding was provided by Biologic Environmental Survey Pty Ltd. and the eDNA for Global Biodiversity (eDGES) program.

## Ethics Statement

The authors have nothing to report.

## Conflicts of Interest

During the preparation of this paper, M.R., C.H., J.D., and J.H. were employed by Biologic Environmental Survey Pty Ltd.

## Supporting information


**Table S1:** List of sites where sediment was collected at Lake Way for the two experiments carried out in this study.
**Table S2:** Custom COI and 16S sequences generated from Lake Way as part of this study.
**Table S3:** Summary of reads after each filtering stage for both experiments and assays utilised in this study. Data for “eDNAFlow”, “Unassigned reads”, “Decontamination” and “Non‐target” are presented as reads (unique ZOTUs). The “Non‐target removed” is the reads and ZOTUs present in the final dataset. Mean read depth ± standard error and number of samples (*n* = X) in the final dataset of each assay for either experiment.
**Table S4:** List of taxa detected using fwh and Crust16S metabarcoding assays with samples processed by sugar‐flotation (Sugar) and standard sediment DNA extraction (Sediment) in Experiment 1. Numbers reflect the number of biological replicates that a species was detected in at a site.
**Table S5:** Summary of the mean number of reads per sample and the number of samples which passed all data filtering steps split by assay and the four sediment weight categories tested in Experiment 2.
**Table S6:** Taxa detected in Experiment 2 split by assay (Crust16S, fwh), sediment weight class and site. Numbers reflect the number of biological replicates that a species was detected in at a site.
**Table S7:** Abiotic data collected during the rehydration trials for Experiment 2. Wetting event refers to either the initial wetting cycling (W1) or the second round of re‐wetting (W2). EC = electrical conductivity and DO = dissolved oxygen.

## Data Availability

All raw sequencing data and associated metadata that support the findings of this study are publicly available at Dryad Digital Repository: http://10.5061/dryad.stqjq2chp.
