# Supplementary material for: Desiccation to DNA: Bulk DNA Metabarcoding of Macroinvertebrate Egg Banks via Sugar‐Flotation Enhances Biodiversity Surveys of Dry Salt Lakes
Source: Ecol Evol. 2026 Aug 21;16(8):e74229. doi: 10.1002/ece3.74229 (PMC13498703; doi:10.1002/ece3.74229)
Supplement: Supplementary file 1 [file ECE3-16-e74229-s001.docx]

Supplementary Table 1: List of sites where sediment was collected at Lake Way for the two experiments carried out in this study.

| Site | Date | Latitude | Longitude |
| --- | --- | --- | --- |
| Experiment 1 |  |  |  |
| Site 1 | 1/11/2023 | -26.7759 | 120.2953 |
| Site 2 | 1/11/2023 | -26.8218 | 120.3516 |
| Site 3 | 1/11/2023 | -26.7303 | 120.3724 |
| Site 4 | 1/11/2023 | -26.7764 | 120.3990 |
| Site 5 | 1/11/2023 | -26.8189 | 120.3767 |
| Site 6 | 1/11/2023 | -26.8653 | 120.4223 |
| Experiment 2 |  |  |  |
| Site 7 | 27/11/2024 | -26.7550 | 120.3940 |
| Site 2 | 28/11/2024 | -26.8218 | 120.3516 |
| Site 3 | 27/11/2024 | -26.7303 | 120.3724 |
| Site 5 | 28/11/2024 | -26.8189 | 120.3767 |

Supplementary Table 2: Custom *COI* and *16S* sequences generated from Lake Way as part of this study.

| **Gene** | **Accession numbers** | **Taxa** |
| --- | --- | --- |
| 16S | PZ048039 | Triops sp. BV-2012 |
| 16S | PZ027474 | Daphnia queenslandensis |
| 16S | PZ027475 | Daphnia queenslandensis |
| 16S | PZ027476 | Daphnia muddensis |
| 16S | PZ027477 | Reticypris `sp. Biologic-OSTR106` |
| 16S | PZ027478 | Patcypris outback |
| 16S | PZ027479 | Reticypris `sp. Biologic-OSTR106` |
| 16S | PZ027480 | Branchinella nichollsi |
| 16S | PZ027481 | Branchinella nichollsi |
| 16S | PZ027482 | Ozestheria sp. lineage T |
| 16S | PZ027483 | Eocyzicus careyensis |
| 16S | PZ027484 | Eocyzicus armatus |
| 16S | PZ048040 | Parartemia laticaudata |
| 16S | PZ048041 | Patcypris outback |
| 16S | PZ048042 | Patcypris outback |
| 16S | PZ048043 | Patcypris outback |
| COI | PZ037862 | Cyprididae `sp. LiWA-OST002` |
| COI | PZ037863 | Cyprididae `sp. LiWA-OST002` |
| COI | PZ037864 | Reticypris `sp. Biologic-OSTR106` |
| COI | PZ037865 | Reticypris `sp. Biologic-OSTR106` |
| COI | PZ037866 | Diacypris `sp. LiWA-OST001` |
| COI | PZ037867 | Diacypris `sp. LiWA-OST001` |
| COI | PZ037868 | Diacypris `sp. LiWA-OST001` |
| COI | PZ037869 | Cypretta `sp. Biologic-OSTR017` |
| COI | PZ037871 | Apocyclops `sp. Biologic-CYCL092` |
| COI | PZ037872 | Meridiecyclops `sp. Biologic-CYCL093` |
| COI | PZ037873 | Schizopera `sp. Biologic-HARP080` |
| COI | PZ037874 | Daphnia queenslandensis |
| COI | PZ037875 | Daphnia queenslandensis |
| COI | PZ037876 | Daphnia muddensis |
| COI | PZ037877 | Reticypris `sp. Biologic-OSTR106` |
| COI | PZ037878 | Patcypris outback |
| COI | PZ037879 | Patcypris outback |
| COI | PZ037880 | Reticypris `sp. Biologic-OSTR106` |
| COI | PZ037881 | Branchinella nichollsi |
| COI | PZ037882 | Branchinella nichollsi |
| COI | PZ037883 | Ozestheria sp. lineage T |
| COI | PZ037884 | Eocyzicus careyensis |
| COI | PZ037885 | Eocyzicus armatus |
| COI | PZ039270 | Parartemia laticaudata |
| COI | PZ039271 | Patcypris outback |
| COI | PZ039272 | Patcypris outback |
| COI | PZ039273 | Patcypris outback |

Supplementary Table 3: Summary of reads after each filtering stage for both experiments and assays utilised in this study. Data for “eDNAFlow”, “Unassigned reads”, “Decontamination” and “Non-target” are presented as reads (unique ZOTUs). The “Non-target removed” is the reads and ZOTUs present in the final dataset. Mean read depth ± standard error and number of samples (n = X) in the final dataset of each assay for either experiment.

| Assay | Post eDNAFlow | Unassigned reads removed | Decontamination | Non-target taxa removed | Mean read depth |
| --- | --- | --- | --- | --- | --- |
| Experiment 1 |  |  |  |  |  |
| fwh | 10,134,993 (996) | 3,423,638 (159) | 3,031,286 (157) | 2,738,487 (23) | 152,138 ± 35,411 (n = 17) |
| Crust16S | 8,202,821 (72) | 8,193,206 (48) | 8,154,885 (11) | 7,447,950 (8) | 827,550 ± 235,282 (n = 9) |
| Experiment 2 |  |  |  |  |  |
| fwh | 37,049,958 (1716) | 16,168,053 (482) | 16,012,850 (481) | 14,547,983 (151) | 323,289 ± 22,547 (n = 45) |
| Crust16S | 25,897,554 (152) | 23,685,380 (77) | 23,518,916 (70) | 23,391,626 (43) | 668,332 ± 72,283 (n = 35) |

Supplementary Table 4: List of taxa detected using fwh and Crust16S metabarcoding assays with samples processed by sugar-flotation (Sugar) and standard sediment DNA extraction (Sediment) in Experiment 1. Numbers reflect the number of biological replicates that a species was detected in at a site.

| **Taxa** | **Crust16S** | | **fwh** | |
| --- | --- | --- | --- | --- |
|  | **Sediment** | **Sugar** | **Sediment** | **Sugar** |
| **Branchiopoda** |  |  |  |  |
| **Anostraca** |  |  |  |  |
| Parartemiidae |  |  |  |  |
| ***Parartemia*** |  |  |  |  |
| *Parartemia laticaudata* |  |  |  |  |
| Site 2 |  | 2 |  | 1 |
| Site 3 |  | 3 |  | 2 |
| Site 4 |  | 1 |  | 1 |
| Thamnocephalidae |  |  |  |  |
| ***Branchinella*** |  |  |  |  |
| *Branchinella nichollsi* |  |  |  |  |
| Site 3 |  | 1 |  |  |
| *Branchinella* sp. |  |  |  |  |
| Site 3 |  | 1 |  |  |
| **Diplostraca** |  |  |  |  |
| Cyzicidae |  |  |  |  |
| ***Eocyzicus*** |  |  |  |  |
| *Eocyzicus careyensis* |  |  |  |  |
| Site 2 |  | 2 |  |  |
| Site 4 |  | 1 |  |  |
| Site 6 |  | 1 |  |  |
| **Notostraca** |  |  |  |  |
| Triopsidae |  |  |  |  |
| ***Triops*** |  |  |  |  |
| *Triops* sp. BV-2012 |  |  |  |  |
| Site 1 |  |  |  | 1 |
| Site 2 |  |  |  | 2 |
| Site 4 |  | 1 |  | 1 |
| **Maxillipoda** |  |  |  |  |
| **Harpacticoida** |  |  |  |  |
| Miraciidae |  |  |  |  |
| ***Schizopera*** |  |  |  |  |
| *Schizopera* `sp. Biologic-HARP080` |  |  |  |  |
| Site 1 |  |  |  | 1 |
| **Ostracoda** |  |  |  |  |
| **Podocopida** |  |  |  |  |
| Cyprididae |  |  |  |  |
| ***Patcypris*** |  |  |  |  |
| *Patcypris outback* |  |  |  |  |
| Site 1 |  |  |  | 3 |
| Site 2 |  |  | 1 | 3 |
| Site 3 |  |  |  | 3 |
| Site 4 |  |  |  | 2 |
| Site 5 |  |  |  | 3 |
| Site 6 |  |  |  | 3 |
| ***Reticypris*** |  |  |  |  |
| *Reticypris* `sp. Biologic-OSTR106` |  |  |  |  |
| Site 1 |  |  |  | 2 |

Supplementary Table 5: Summary of the mean number of reads per sample and the number of samples which passed all data filtering steps split by assay and the four sediment weight categories tested in Experiment 2.

| Assay | Mean read depth ± SE | N samples |
| --- | --- | --- |
| fwh2 |  |  |
| 50 | 162,604 ± 27,404 | 9 |
| 250 | 300,128 ± 43,588 | 11 |
| 500 | 433,247 ± 50,244 | 12 |
| 1000 | 382,014 ± 42,624 | 12 |
| Crust16S |  |  |
| 50 | 380,482 ± 238,512 | 4 |
| 250 | 185,172 ± 80,252 | 9 |
| 500 | 179,137 ± 60,062 | 11 |
| 1000 | 295,553 ± 105,357 | 11 |

Supplementary Table 6: Taxa detected in Experiment 2 split by assay (Crust16S, fwh), sediment weight class and site. Numbers reflect the number of biological replicates that a species was detected in at a site.

| **Taxa** | **Crust16S** | | | | **fwh** | | | |
| --- | --- | --- | --- | --- | --- | --- | --- | --- |
|  | **50** | **250** | **500** | **1000** | **50** | **250** | **500** | **1000** |
| **Branchiopoda** |  |  |  |  |  |  |  |  |
| **Anostraca** |  |  |  |  |  |  |  |  |
| Parartemiidae |  |  |  |  |  |  |  |  |
| ***Parartemia*** |  |  |  |  |  |  |  |  |
| *Parartemia laticaudata* |  |  |  |  |  |  |  |  |
| Site 7 |  | 1 | 2 | 3 |  | 1 | 2 | 3 |
| Site 2 |  |  |  | 2 |  |  |  | 2 |
| Site 3 |  |  | 1 | 2 |  |  | 1 |  |
| Site 4 |  |  |  | 1 |  |  |  | 1 |
| Thamnocephalidae |  |  |  |  |  |  |  |  |
| ***Branchinella*** |  |  |  |  |  |  |  |  |
| *Branchinella australiensis* |  |  |  |  |  |  |  |  |
| Site 7 |  |  |  | 1 |  |  |  |  |
| *Branchinella nichollsi* |  |  |  |  |  |  |  |  |
| Site 7 | 2 | 3 | 3 | 3 | 2 | 3 | 3 | 3 |
| Site 2 |  |  |  | 1 |  |  |  |  |
| Site 3 |  | 3 | 2 | 3 |  | 3 | 2 | 2 |
| Site 4 |  |  | 2 |  |  |  | 2 |  |
| *Branchinella* sp. |  |  |  |  |  |  |  |  |
| Site 7 | 2 | 3 | 3 | 3 |  | 1 |  |  |
| Site 3 |  | 2 |  | 1 |  |  |  |  |
| Site 4 |  |  | 1 |  |  |  |  |  |
| **Diplostraca** |  |  |  |  |  |  |  |  |
| Eocyzicidae |  |  |  |  |  |  |  |  |
| *Eocyzicus* |  |  |  |  |  |  |  |  |
| *Eocyzicus armatus* |  |  |  |  |  |  |  |  |
| Site 7 |  |  |  |  |  | 1 | 2 | 3 |
| Site 2 |  |  |  |  |  |  | 1 | 1 |
| Site 3 |  |  |  |  |  | 1 |  |  |
| Site 4 |  |  |  |  |  | 2 | 2 | 3 |
| *Eocyzicus careyensis* |  |  |  |  |  |  |  |  |
| Site 2 |  |  | 1 | 1 |  |  |  |  |
| Site 4 |  | 2 | 3 | 3 |  | 1 | 2 | 2 |
| *Eocyzicus* sp. |  |  |  |  |  |  |  |  |
| Site 7 |  | 1 | 1 |  |  |  |  |  |
| Site 2 |  |  | 1 | 1 |  |  |  |  |
| Site 3 |  | 1 |  |  |  |  |  |  |
| Site 4 | 1 | 2 | 2 | 3 |  |  |  |  |
| ***Ozestheria*** |  |  |  |  |  |  |  |  |
| *Ozestheria beleriandensis* |  |  |  |  |  |  |  |  |
| Site 4 |  |  |  | 1 |  |  |  |  |
| *Ozestheria matuwa* |  |  |  |  |  |  |  |  |
| Site 7 |  | 1 | 2 | 1 |  | 1 | 2 | 1 |
| *Ozestheria sarsii* |  |  |  |  |  |  |  |  |
| Site 7 | 2 | 3 | 3 | 3 | 2 | 3 | 3 | 3 |
| Site 3 |  | 1 |  |  |  | 1 |  |  |
| Site 4 |  |  |  | 1 |  |  |  | 1 |
| *Ozestheria* sp. |  |  |  |  |  |  |  |  |
| Site 7 | 1 | 1 | 2 | 1 | 2 | 3 | 2 | 3 |
| Macrothricidae |  |  |  |  |  |  |  |  |
| Macrothricidae sp. |  |  |  |  |  |  |  |  |
| Site 7 |  |  |  |  |  |  | 1 |  |
| Site 3 |  |  |  |  |  | 1 | 1 |  |
| Daphniidae |  |  |  |  |  |  |  |  |
| ***Daphnia*** |  |  |  |  |  |  |  |  |
| *Daphnia muddensis* |  |  |  |  |  |  |  |  |
| Site 7 |  |  |  |  | 1 |  | 3 | 3 |
| Site 3 |  |  |  |  |  | 1 | 2 |  |
| *Daphnia queenslandensis* |  |  |  |  |  |  |  |  |
| Site 7 |  |  |  |  |  | 3 | 1 | 2 |
| Site 2 |  |  |  |  | 3 | 2 | 3 | 2 |
| Site 3 |  |  |  |  |  |  | 1 | 1 |
| Site 4 |  |  |  |  |  | 1 | 1 | 3 |
| **Notostraca** |  |  |  |  |  |  |  |  |
| Triopsidae |  |  |  |  |  |  |  |  |
| ***Triops*** |  |  |  |  |  |  |  |  |
| *Triops* sp. BV-2012 |  |  |  |  |  |  |  |  |
| Site 7 |  |  |  |  |  | 3 | 3 | 3 |
| Site 2 |  | 1 | 1 |  |  | 1 | 3 | 2 |
| Site 3 |  |  |  |  |  |  |  | 1 |
| Site 4 |  |  |  |  |  | 2 | 1 | 3 |
| **Copepoda** |  |  |  |  |  |  |  |  |
| **Calanoida** |  |  |  |  |  |  |  |  |
| Centropagidae |  |  |  |  |  |  |  |  |
| **Boeckella** |  |  |  |  |  |  |  |  |
| *Boeckella triarticulata* |  |  |  |  |  |  |  |  |
| Site 7 |  |  |  |  |  |  |  | 1 |
| Site 4 |  |  |  |  |  |  | 1 | 2 |
| Copepoda `sp. Biologic-CALA001` |  |  |  |  |  |  |  |  |
| Site 2 |  |  |  |  |  |  |  | 1 |
| **Cyclopoida** |  |  |  |  |  |  |  |  |
| Cyclopidae |  |  |  |  |  |  |  |  |
| ***Meridiecyclops*** |  |  |  |  |  |  |  |  |
| *Meridiecyclops* `sp. Biologic-CYCL093` |  |  |  |  |  |  |  |  |
| Site 7 |  |  |  |  |  | 1 |  |  |
| **Ostracoda** |  |  |  |  |  |  |  |  |
| **Podocopida** |  |  |  |  |  |  |  |  |
| Cyprididae |  |  |  |  |  |  |  |  |
| ***Diacypris*** |  |  |  |  |  |  |  |  |
| *Diacypris* `sp. LiWA-OST001` |  |  |  |  |  |  |  |  |
| Site 7 |  |  |  |  |  |  | 1 | 2 |
| Site 2 |  |  |  |  | 3 | 2 | 3 | 2 |
| Site 3 |  |  |  |  |  | 3 | 1 | 2 |
| ***Patcypris*** |  |  |  |  |  |  |  |  |
| *Patcypris outback* |  |  |  |  |  |  |  |  |
| Site 7 |  |  |  |  | 3 | 3 | 3 | 3 |
| Site 2 |  |  |  |  | 3 | 2 | 3 | 3 |
| Site 3 |  |  |  |  | 1 | 3 | 3 | 3 |
| Site 4 |  |  |  |  | 2 | 3 | 3 | 3 |
| ***Repandocypris*** |  |  |  |  |  |  |  |  |
| *Repandocypris austinensis* |  |  |  |  |  |  |  |  |
| Site 7 |  |  |  |  |  | 2 | 1 | 1 |
| Site 2 |  |  |  |  |  | 2 | 3 | 1 |
| Site 3 |  |  |  |  |  | 1 |  |  |
| ***Reticypris*** |  |  |  |  |  |  |  |  |
| *Reticypris* `sp. Biologic-OSTR106` |  |  |  |  |  |  |  |  |
| Site 7 |  |  |  |  | 1 | 3 | 3 | 3 |
| Site 2 |  |  |  |  | 3 | 2 | 3 | 2 |
| Site 3 |  |  |  |  |  | 3 | 2 | 3 |
| Cyprididae `sp. LiWA-OST002` |  |  |  |  |  |  |  |  |
| Site 7 |  |  |  |  | 3 | 3 | 3 | 3 |
| Site 3 |  |  |  |  |  | 1 | 3 | 1 |
| Site 4 |  |  |  |  |  |  |  | 1 |
| Cyprididae sp. |  |  |  |  |  |  |  |  |
| Site 7 |  |  |  |  | 3 | 3 | 3 | 3 |
| Site 3 |  |  |  |  |  |  | 1 |  |
| **Insecta** |  |  |  |  |  |  |  |  |
| **Diptera** |  |  |  |  |  |  |  |  |
| Ceratopogonidae |  |  |  |  |  |  |  |  |
| ***Nilobezzia*** |  |  |  |  |  |  |  |  |
| *Nilobezzia* sp. |  |  |  |  |  |  |  |  |
| Site 7 |  |  |  |  |  | 1 | 3 | 3 |
| Chironomidae |  |  |  |  |  |  |  |  |
| ***Tanytarsus*** |  |  |  |  |  |  |  |  |
| *Tanytarsus barbitarsis* |  |  |  |  |  |  |  |  |
| Site 7 |  |  |  |  | 2 | 2 | 3 | 3 |
| Site 2 |  |  |  |  |  |  | 2 | 2 |
| *Tanytarsus bispinosus* |  |  |  |  |  |  |  |  |
| Site 7 |  |  |  |  |  |  |  | 2 |

Supplementary Table 7: Abiotic data collected during the rehydration trials for Experiment 2. Wetting event refers to either the initial wetting cycling (W1) or the second round of re-wetting (W2). EC = electrical conductivity and DO = dissolved oxygen.

| Site | Wetting event | Date | pH | Temp (ºC) | EC (µs/cm) | DO mg/l |
| --- | --- | --- | --- | --- | --- | --- |
| Site 3 | W1 | 24/03/2025 | 8.17 | 25.4 | 5341 | 6.24 |
| Site 3 | W1 | 26/03/2025 | 8.18 | 25.4 | 5526 | - |
| Site 3 | W1 | 31/03/2025 | 8.1 | 23.26 | 5774 | 8.32 |
| Site 3 | W1 | 9/04/2025 | 7.94 | 22.2 | 8703 | 8.95 |
| Site 3 | W1 | 11/04/2025 | 8.23 | 24.9 | 6116 | 5.99 |
| Site 3 | W1 | 14/04/2025 | 8.28 | 25.1 | 6195 | 5.53 |
| Site 3 | W1 | 16/04/2025 | 8.04 | 23.2 | 6603 | 9.36 |
| Site 3 | W2 | 7/05/2025 | 7.7 | 23.2 | 1091 | 6.35 |
| Site 3 | W2 | 9/05/2025 | 7.73 | 23.33 | 1390 | 8.8 |
| Site 3 | W2 | 12/05/2025 | 7.38 | 25.26 | 2643 | 7.62 |
| Site 3 | W2 | 14/05/2025 | 7.81 | 24.9 | 1911 | 7.88 |
| Site 3 | W2 | 16/05/2025 | 7.7 | 23.7 | 1979 | 8.51 |
| Site 3 | W2 | 19/05/2025 | 7.68 | 23.5 | 3083 | 7.43 |
| Site 3 | W2 | 21/05/2025 | 7.87 | 23.4 | 2141 | 8.17 |
| Site 3 | W2 | 23/05/2025 | 7.47 | 22.7 | 2185 | 8.58 |
| Site 3 | W2 | 26/05/2025 | 7.89 | 20.7 | 2127 | 10.22 |
| Site 3 | W2 | 28/05/2025 | 7.93 | 20.5 | 2137 | - |
| Site 4 | W1 | 24/03/2025 | 8.11 | 25.7 | 9922 | 6.13 |
| Site 4 | W1 | 26/03/2025 | 8.09 | 25.7 | 10056 | - |
| Site 4 | W1 | 31/03/2025 | 8.01 | 23.41 | 10650 | 8.15 |
| Site 4 | W1 | 9/04/2025 | 8.02 | 22.3 | 9994 | 8.33 |
| Site 4 | W1 | 11/04/2025 | 8.26 | 25.2 | 10681 | 5.79 |
| Site 4 | W1 | 14/04/2025 | 8.3 | 25.3 | 10742 | 5.37 |
| Site 4 | W1 | 16/04/2025 | 8.01 | 23.3 | 10411 | 9.09 |
| Site 4 | W2 | 7/05/2025 | 7.73 | 23.3 | 1445 | 6.59 |
| Site 4 | W2 | 9/05/2025 | 7.62 | 23.5 | 1781 | 8.8 |
| Site 4 | W2 | 12/05/2025 | 7.59 | 25.54 | 3122 | 7.68 |
| Site 4 | W2 | 14/05/2025 | 7.82 | 25.1 | 2218 | 7.64 |
| Site 4 | W2 | 16/05/2025 | 7.78 | 23.9 | 2260 | 8.49 |
| Site 4 | W2 | 19/05/2025 | 7.89 | 23.7 | 3496 | 7.73 |
| Site 4 | W2 | 21/05/2025 | 7.9 | 23.4 | 2383 | 8 |
| Site 4 | W2 | 23/05/2025 | 7.7 | 22.92 | 2471 | 8.6 |
| Site 4 | W2 | 26/05/2025 | 7.89 | 21 | 2350 | 9.86 |
| Site 4 | W2 | 28/05/2025 | 7.92 | 20.7 | 2336 | - |
| Site 7 | W1 | 24/03/2025 | 7.82 | 25.6 | 6140 | 5.41 |
| Site 7 | W1 | 26/03/2025 | 7.95 | 25.6 | 6265 | - |
| Site 7 | W1 | 31/03/2025 | 8.1 | 23.18 | 6572 | 8.03 |
| Site 7 | W1 | 9/04/2025 | 8 | 22.2 | 6396 | 8.26 |
| Site 7 | W1 | 11/04/2025 | 8.26 | 25.1 | 6840 | 5.72 |
| Site 7 | W1 | 14/04/2025 | 8.29 | 25.1 | 6941 | 5.01 |
| Site 7 | W1 | 16/04/2025 | 8.03 | 23.1 | 6701 | 8.58 |
| Site 7 | W2 | 7/05/2025 | 7.59 | 23.2 | 952 | 6.33 |
| Site 7 | W2 | 9/05/2025 | 7.62 | 23.33 | 1080 | 8.35 |
| Site 7 | W2 | 12/05/2025 | 7.69 | 25.11 | 2143 | 7.37 |
| Site 7 | W2 | 14/05/2025 | 7.87 | 24.7 | 1697 | 7.54 |
| Site 7 | W2 | 16/05/2025 | 7.82 | 23.6 | 1805 | 8.55 |
| Site 7 | W2 | 19/05/2025 | 7.93 | 23.5 | 2882 | 7.93 |
| Site 7 | W2 | 21/05/2025 | 7.96 | 23.3 | 2001 | 7.94 |
| Site 7 | W2 | 23/05/2025 | 7.98 | 23.1 | 2078 | 8.54 |
| Site 7 | W2 | 26/05/2025 | 7.92 | 21.2 | 2037 | 9.39 |
| Site 7 | W2 | 28/05/2025 | 7.91 | 20.9 | 2022 | - |
| Site 2 | W1 | 24/03/2025 | 8.06 | 25.5 | 9640 | 5.87 |
| Site 2 | W1 | 26/03/2025 | 7.97 | 25.4 | 9756 | - |
| Site 2 | W1 | 31/03/2025 | 7.99 | 23.06 | 10170 | 8.23 |
| Site 2 | W1 | 9/04/2025 | 7.94 | 22.1 | 9596 | 8.69 |
| Site 2 | W1 | 11/04/2025 | 8.07 | 24.3 | 10110 | 5.81 |
| Site 2 | W1 | 14/04/2025 | 8.1 | 24.2 | 10257 | 5.13 |
| Site 2 | W1 | 16/04/2025 | 7.91 | 22.7 | 10106 | 9.26 |
| Site 2 | W2 | 7/05/2025 | 7.55 | 23 | 1093 | 6.31 |
| Site 2 | W2 | 9/05/2025 | 7.76 | 23.16 | 1512 | 8.78 |
| Site 2 | W2 | 12/05/2025 | 7.59 | 24.13 | 2811 | 7.31 |
| Site 2 | W2 | 14/05/2025 | 7.83 | 23.4 | 2099 | 7.84 |
| Site 2 | W2 | 16/05/2025 | 7.79 | 22.9 | 2230 | 8.75 |
| Site 2 | W2 | 19/05/2025 | 7.85 | 23.2 | 3518 | 8 |
| Site 2 | W2 | 21/05/2025 | 7.92 | 23.3 | 2427 | 7.91 |
| Site 2 | W2 | 23/05/2025 | 7.97 | 22.9 | 2511 | 8.56 |
| Site 2 | W2 | 26/05/2025 | 7.86 | 21.2 | 2399 | 9.22 |
| Site 2 | W2 | 28/05/2025 | 7.9 | 20.9 | 2382 | - |
